# Neurology

**Published:** 1904-11-12

**Authors:** 


### NEUROLOGY.

**Multiple Sclerosis.**—In a paper by Jeliffe[1] the statistics of the Vanderbilt Clinik in New York during some sixteen years have been examined to see whether any light is shown on the etiology of this disease. It may be remarked that the diagnosis was not made without the presence of at least three of the four cardinal symptoms—increased reflexes, intention tremor, nystagmus, and scanning speech. The incidence of the disease was about 1 in 300 of all neurological cases. It is therefore far less common in the United States than in England, where the proportion has been estimated, by Bramwell and Williamson, at from 1·7 to 2·2 per cent. of neurological cases. Sex plays but little part in the etiology of the affection. The youngest patient was aged 4 years, the oldest 68, but the majority occurred between 20 and 60 years. The type of occupation and heredity play no important *rôle*. Acute infectious disease is an important etiological factor, but not the only one, as Marie supposed. It occurred in 25 per cent. of the cases. Typhoid, malaria, rheumatism, small-pox, and pneumonia are the commonest antecedents. Trauma occurred in 12 per cent. The influence of poisons was of very minor importance. A clinical account of the disease has been given in a lecture by Bramwell,[2] who says that the features presented are very diverse, due partly to the multiplicity of the lesions, partly to the fact that the symptoms vary in a most remarkable way from time to time, and partly to the great variation in the duration of the cases. The early stages are often thought to be hysterical, or the disease may be diagnosed as ataxic paraplegia, or as disseminated myelitis, or syphilitic myelitis, or general paralysis. The affection is particularly apt to attack people in early adult life, and, much more frequently, comparatively well-to-do people than the lower classes, *e.g.* women engaged in household work—not servants, but single girls and married women living at home. Preceding infectious disease, so often thought to be a cause, is frequently found, on careful questioning, to be merely an aggravating one. For instance, a woman dated her disease from an attack of influenza three years before, but had had diplopia seven years previously. The great majority of patients have been perfectly well, strong, and healthy young men and women until the affection developed, and they do not present any stigmata of degeneration. The onset of the disease is often insidious—frequently temporary numbness or weakness, which, after lasting a month or two, disappears. Diplopia is common, also giddiness. Ocular paralysis, with strabismus, often temporary in duration, is common. In a suspected case of disseminated sclerosis the presence of the Argyll-Robertson pupil usually indicates previous syphilis and suggests that the case is really one of general paralysis. Vision is affected in at least 50 per cent. of cases; this affection is sometimes transitory, sometimes permanent, usually bilateral though sometimes unilateral. Rapid or sudden impairment of sight, rapidly recovered from after lasting a longer or shorter time, though usually believed to be hysterical, is generally due to disseminated sclerosis. Retrobulbar neuritis occurs in a certain proportion of cases, and may be an initial symptom. It is a remarkable fact that the visual condition and the ophthalmoscopic appearance of the optic discs rarely correspond. The optic atrophy is usually slight and very rarely progresses to complete blindness. Optic neuritis is very rare. In differentiating disseminated sclerosis and hysteria the Babinski sign is of the utmost importance. Though other symptoms, such as loss of power, numbness, giddiness, diplopia, impairment of vision, volitional tremor, may for a time disappear, the Babinski sign remains, indicating the presence of organic disease. The majority of cases are chronic, with repeated exacerbations and remissions. Now many authorities think that the patches of sclerosis, which are the pathological substratum of the disease, result from the irritation produced by some toxin carried to the nervous tissue. If this is so, the process is probably one of auto-intoxication, for it is difficult to suppose fresh doses of the poison to be introduced from without during a long period of years. Though remissions may last a long time, it is doubtful if a permanent arrest or cure ever actually occurs. Treatment is very unsatisfactory. Strychnine is in many cases harmful, though it may be useful in a very few exceptional cases where there is muscular atrophy and diminution or loss of the deep reflexes. In the early stages electricity is often beneficial, more particularly in dealing with functional symptoms. Buzzard [3] has also published a clinical lecture on insular sclerosis, in which the fact of remissions and relapses is emphasised, and from which the same deduction is drawn, viz., that the disease is of exogenous origin. After citing several cases in which this occurred, he states that the most likely view is that the essential cause is the presence in the blood-vessels of an infective agent which sets up an inflammatory process in the interstitial tissue. The grossness of the anatomical findings, the absence of any family history of a like affection, and the want of any slow and comparatively steady progress, are against the theory that the disease is of endogenous origin. In these respects the disease is similar to syphilitic affections of the nervous system.

**Trauma in Relation to Disease of the Nervous System.**—In an address to the Manchester Medical Society, Bury [4] discussed the question as to what extent trauma might be considered an exciting cause of serious nervous disease, and the effects of concussion of the brain and spinal cord apart from the results of injury or disease of their bony envelopes. A man receives a blow on the head which, without seriously damaging the skull, is severe enough to give a violent shake to the intracranial contents. He may or may not lose consciousness, and in either case may make a complete recovery or become the subject of either temporary or permanent symptoms. These symptoms may be clinically classified as (1) Early symptoms indicating meningeal hæmorrhage; (2) symptoms which are not usually conspicuous until some weeks later—viz., neurasthenia, insanity, brain tumour; (3) symptoms of the condition known as "late apoplexy." Meningeal hæmorrhage may be either subcranial or subdural. In the former three clinical stages may be distinguished: an initial stage, in which there is partial or complete loss of consciousness; a lucid interval, lasting from a few hours to a couple of days; and a third stage in which there is stupor, deepening into coma, with spasms, followed by paralysis of the opposite side of the body. The subdural variety is distinguished from the subcranial variety by the absence of a lucid interval and by the more rapid onset and development of compression symptoms, which are more general in distribution. But to these rules there are many exceptions. Two groups of neurasthenia occurring after concussion of the brain may be recognised, one closely resembling ordinary non-traumatic neurasthenia, the other comprising cases where from the first there are signs of cortical lesions The latter class are dull and apathetic, with a tendency to drowsiness and stupor, with loss of memory and other signs of mental deterioration. It is to be noted that this physical and mental incapacity may often follow even slight injuries to the head. The symptoms are thus very different from those of ordinary neurasthenia, where the patient suffers from insomnia, is able to commence work and do it accurately for a short time, but soon tires, lacks concentration, finds his memory unreliable, exhibits indecision in his actions, and is constantly restless and anxious about his condition. There is abundant evidence that insanity may occur after head injuries, and sometimes apparently without evidence of hereditary predisposition. Tumours may owe their origin to nutritive changes produced by concussion. By "late apoplexy" is meant apoplexy, dependent on rupture of a cerebral artery, occurring some few weeks or so after concussion. It would seem that in some cases an accident may be responsible for the arterial disease; thus, the patient may be under 40, and have no signs of heart-disease, arterial deterioration, syphilis, alcoholism, or nephritis. Concussion of the spinal cord—*i.e.* a violent shaking, unaccompanied by direct wounds, fractures, or dislocations—may cause disease, generally of the nature of a spastic paralysis. Those cases which develop early are generally due to hæmorrhage. It is interesting to notice that a few cases of complete paralysis have been recorded in which no lesion of the cord has been discoverable either by the naked eye or the microscope. The assumption is that the nutrition of the nerve elements was abolished. Though concussion may initiate the development of the characteristic signs of tabes, it rarely acts as the sole cause of this disease. It doubtless often co-operates with other causes, especially syphilis, and tends to light up disease which was pre-existent, or for which the ground was duly prepared. Injury to the periphery of the body may cause disease of the spinal cord; thus arthritic changes may lead to muscular atrophy. This may become permanent, and many cases of progressive muscular atrophy appear to originate from a severe sprain or other injury to a limb.

[1] Jour. of Nerv. and Ment. Dis., July. [2] Clin. Jour., June 22. [3] Lancet, July 16. [4] Brit. Med. Jour., April 30.

